# The Dual Effect of the BMP9–ALK1 Pathway in Blood Vessels: An Opportunity for Cancer Therapy Improvement?

**DOI:** 10.3390/cancers13215412

**Published:** 2021-10-28

**Authors:** Blanca Ayuso-Íñigo, Lucía Méndez-García, Miguel Pericacho, José M. Muñoz-Félix

**Affiliations:** 1 Department of Physiology and Pharmacology, University of Salamanca, 37007 Salamanca, Spain; blancayuso97@usal.es (B.A.-Í.); luciamengar@usal.es (L.M.-G.); pericacho@usal.es (M.P.); 2 Group of Pathophysiology of the Vascular Endothelium (ENDOVAS), Institute of Biomedical Research of Salamanca (IBSAL), 37007 Salamanca, Spain; 3 Department of Biochemistry and Molecular Biology, University of Salamanca, 37007 Salamanca, Spain

**Keywords:** tumor angiogenesis, bone morphogenetic protein 9, ALK1, cancer therapy

## Abstract

**Simple Summary:**

The modulation of tumor blood vessels is a great opportunity for improving cancer therapies. Understanding the cellular and molecular players that regulate the biology of tumor blood vessels and tumor angiogenesis is necessary for the development of new anti-tumor strategies. Bone morphogenetic protein 9 (BMP9) is a circulating factor with multiple effects in vascular biology through its receptor activin receptor-like kinase 1 (ALK1). In this review, we give an overview of the possible benefits of modulating BMP9–ALK1 functions for cancer therapy improvement.

**Abstract:**

The improvement of cancer therapy efficacy, the extension of patient survival and the reduction of adverse side effects are major challenges in cancer research. Targeting blood vessels has been considered a promising strategy in cancer therapy. Since the tumor vasculature is disorganized, leaky and triggers immunosuppression and tumor hypoxia, several strategies have been studied to modify tumor vasculature for cancer therapy improvement. Anti-angiogenesis was first described as a mechanism to prevent the formation of new blood vessels and prevent the oxygen supply to tumor cells, showing numerous limitations. Vascular normalization using low doses of anti-angiogenic drugs was purposed to overcome the limitations of anti-angiogenic therapies. Other strategies such as vascular promotion or the induction of high endothelial venules are being studied now to improve cancer therapy. Bone morphogenetic protein 9 (BMP9) exerts a dual effect through the activin receptor-like kinase 1 (ALK1) receptor in blood vessel maturation or activation phase of angiogenesis. Thus, it is an interesting pathway to target in combination with chemotherapies or immunotherapies. This review manuscript explores the effect of the BMP9–ALK1 pathway in tumor angiogenesis and the possible usefulness of targeting this pathway in anti-angiogenesis, vascular normalization or vascular promotion therapies.

## 1. Tumor Vascularization

Cancer is not just an isolated and homogenous mass of proliferating malignant cells, but a complex tissue that comprises resident and infiltrating stromal cells, secreted factors and extracellular matrix proteins. The major stromal cell subtypes are cancer-associated fibroblasts (CAFs), endothelial cells (ECs), pericytes and numerous immune cells. The dynamic and bidirectional interactions between malignant cells and non-malignant cells surrounding them create the tumor microenvironment (TME), which affects the development and progression of cancer [1,2,3]. Moreover, TME can also influence the response and resistance to therapies [4], explaining the recent emphasis on targeting different components of the TME and with the immune checkpoint inhibitors or immunogenic chemotherapies being the most revolutionary milestone in this field [5,6,7].

Tumor vasculature comprises an essential part of the TME; in fact, tumors cannot grow beyond a few millimeters without generating a vascular network [8]. Besides supplying oxygen and nutrients and removing catabolic products, tumor blood vessels also act as a road for both the metastatic dissemination and the infiltration of immune cells [9,10]. The main mode of tumor vessel formation is angiogenesis, although other mechanisms exist too, such as intussusceptive angiogenesis and different non-angiogenic mechanisms (like vascular mimicry, recruitment of bone marrow-derived precursor cells or vessel co-option) [9,11]. Thus, differing on the mechanism of tumor vessel formation there are two types of tumors: angiogenic tumors and non-angiogenic tumors.

In contrast to angiogenic tumors, non-angiogenic tumors grow without the formation of new blood vessels and they progress using alternative mechanisms such as vessel co-option or vascular mimicry [12,13]. Tumors that undergo vessel co-option use the preexisting vasculature to grow and disseminate. This mechanism takes place mostly in highly vascularized tissues such as brain, liver or lungs [14]. On the other hand, vascular mimicry is a phenomenon in which tumor cells are differentiated into blood vessel-like structures [13]. In any case, non-angiogenic tumors are in the minority since the vascularization of most solid tumors is formed by angiogenesis.

Angiogenesis is the formation of new blood vessels from pre-existing ones through a highly regulated process controlled by a balance between pro- and antiangiogenic factors [11]. However, when it comes to cancer, this equilibrium is disrupted. Stimulatory angiogenic factors such as vascular endothelial growth factor-A (VEGFA), basic fibroblast growth factor (bFGF), platelet derived growth factor (PDGF), angiopoietins and transforming growth factor (TGF) are ubiquitously abundant in the TME, triggering the angiogenic switch [9,10]. These cytokines are frequently secreted by tumor cells but can also derive from stromal cells, such as regulatory T cells or tumor-associated macrophages [15]. Among them, VEGFA is the pivotal mediator of angiogenesis, acting mainly via the activation of VEGFR2, its receptor, on endothelial cells. The VEGFA/VEGFR2 axis induces EC proliferation, migration, invasion and survival. Not surprisingly, the anti-VEGF monoclonal antibody Bevacizumab was the first anti-angiogenic therapy approved by the FDA, as we will review in detail later [16].

Tumor vasculature, as a relevant part of the TME, can be exploited to control tumor growth and progression. The first vessel modulation strategy was anti-angiogenesis. This approach considered tumor blood vessels as a target and hypothesized that reducing tumor blood vessel density would starve tumors to death [17]. In recent years, different strategies have emerged using the blood vessels as tools to improve cancer therapy. These strategies postulate that the functionality of the vasculature can be modulated to promote a favorable tumor microenvironment or to improve the delivery of drugs.

## 2. Targeting Tumor Vasculature to Improve Cancer Therapy

Targeting blood vessels and the tumor microenvironment has been studied for many years based on the following premises: the tumor needs oxygen and a nutrient supply to grow and to become invasive. On the other hand, scientists and clinicians need to consider the main features in the tumors that can help to treat them better. Tumor blood vessels need to be functional and mature to maintain good blood flow and facilitate good delivery of the therapeutic agent (chemotherapy, immunotherapy) [18,19]. Good oxygenation in the tumors contributes to create an immunopermissive tumor microenvironment, which may provide a better prognosis and outcome [20].

**Figure 1:**
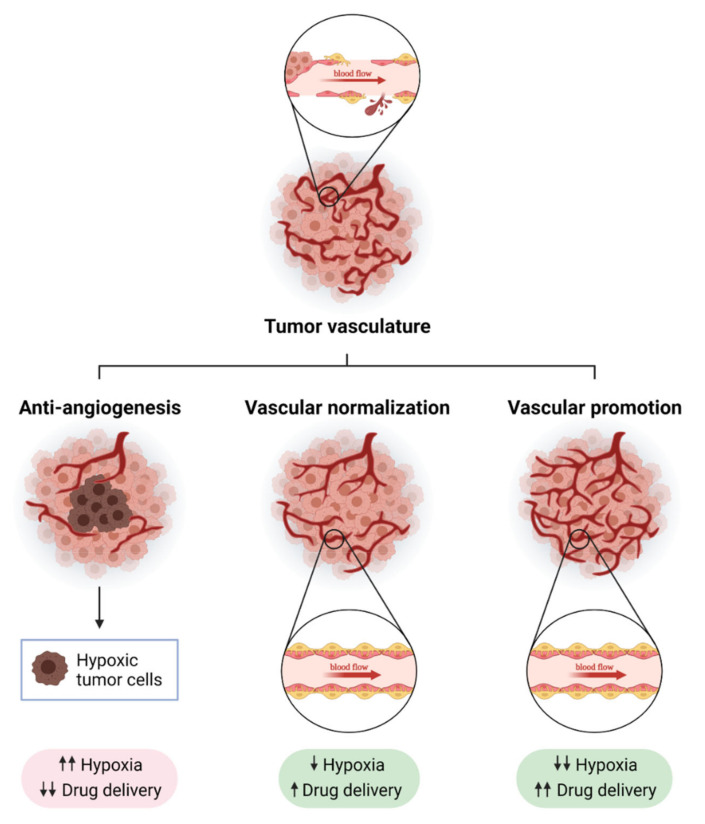
**Strategies to modulate tumor blood vessels to enhance cancer therapy efficacy**. Tumor blood vessels are immature, leaky and show a disorganized structure, which causes hypoxia and immunosuppression (**upper panel**). The modulation of tumor vasculature can enhance anti-tumor strategies. Anti-angiogenesis consists of the inhibition of molecular pathways involved in the promotion of the formation of new blood vessels from pre-existing ones (like VEGF-VEGFR axis, Ang2, or endoglin). A strong reduction in blood vessel density after anti-angiogenic treatment leads to increased tumor hypoxia and poor drug delivery (**bottom left panel**). Normalizing tumor vasculature can overcome some of the limitations of anti-angiogenic therapy: blood flow is increased, which improves drug delivery, and tumors become more oxygenated (**bottom middle panel**). In recent years, a new strategy called “vascular promotion” has been developed using low doses of αvβ3 integrin inhibitors or other molecules such as LPA (lysophosphatidic acid). In contrast to vascular normalization, vascular promotion increases the number of blood vessels promoting an increase in blood flow, and drug delivery and a reduction in tumor hypoxia (**bottom right panel**). Created with BioRender.com, accessed on 17 October 2021.

The main goal of anti-angiogenic therapy is to block the growth of new blood vessels and control tumor growth. For this purpose, a great number of strategies inhibiting VEGF, its receptor VEGFR and other pro-angiogenic molecules were carried out to be used in the clinic, mainly in combination with chemotherapy [21]. However, although some improvements were observed in overall survival, the benefit of anti-angiogenesis is very limited. Excessive pruning of tumor vasculature by using anti-angiogenic drugs leads to hypoxia, resistance and poor drug delivery [22]. Nevertheless, anti-angiogenic drugs can normalize the tumor vasculature, especially when used at a low dose [23,24]. This is the basis of vascular normalization strategies that aim to overcome the limitation of anti-angiogenic therapy, since it can improve blood flow and then tumor oxygenation. One of the main features of vascular normalization is pericyte recruitment. Pericytes are mural cells that support and stabilize tumor blood vessels. Vascular normalization therapies are relevant now, especially because the reduction of hypoxia leads to a favorable tumor microenvironment, which improves the efficacy of current immunotherapies such as immune checkpoint blockade [15]. Oxygenated tumors are normally better infiltrated by CD8^+^ T cells and M1 macrophages while hypoxic tumors attract regulatory T cells and M2 macrophages. Thus, vascular normalization strategies are improving the efficacy of cancer immunotherapy as they are able to transdifferentiate immunosuppressive “cold” tumors into immunopermissive “hot” tumors [25,26,27]. There are also new strategies that aspire to promote tumor blood vessels to improve drug delivery and reduce tumor hypoxia, which are called “vascular promotion” strategies [22] (Figure 1). A good understanding of the cellular and molecular mechanisms involved in tumor blood vessel biology has established numerous strategies targeting some of the “main actors” such as VEGF/VEGFR2 or Ang2/Tie2. However, there are some other molecular players with therapeutic potential that are poorly understood. We will focus this review in the BMP9–ALK1 pathway, which has been studied in depth as an important player of the regulation of vascular homeostasis.

### 2.1. Inhibiting Angiogenesis: “Anti-Angiogenic Therapy”

The concept of “anti-angiogenic therapy” comes from the observations of J. Folkman [8]. Following the observation of the induction of sprouting of new vessels by tumors, it was suggested that inhibition of sprouting angiogenesis could inhibit tumor growth [28]. Later, VEGF was described as a pivotal growth factor involved in EC proliferation during tumor angiogenesis and it was suggested that its inhibition could decrease tumor angiogenesis and consequently tumor growth [17,29,30,31,32,33]. Based on this idea, numerous approaches targeting the VEGF signaling pathway, such as ligand binding agents, receptor targeted suppress antibodies and tyrosine kinase inhibitors (TKIs) have been set up in clinical trials during the last 30 years [28].

Some anti-angiogenic drugs are now approved for cancer treatment. They are used alone or in combination with other therapeutic agents. Most of them are molecules that target VEGF or its receptor VEGFR. Bevacizumab (Avastin) was the first drug approved for the treatment of metastatic colorectal cancer in combination with chemotherapy. Avastin is a humanized anti-VEGFA monoclonal antibody [21]. Secondly, Ranibizumab is a molecule derived from Bevacizumab but having a single antigen-binding site and a higher VEGFA binding activity [21,34]. Moreover, other anti-angiogenic drugs are tyrosine kinase inhibitors that block cell signaling through VEGFR-1, VEGFR-2, PDGFR (PDGF-receptor), FGFR (FGF-receptor) and other signaling pathways [34]. Aflibercept, which is constructed by fusion of VEGF binding domain of both VEGFR1 and VEGFR2, has been approved for metastatic colorectal cancer treatment [35]. Besides the anti-angiogenic agents mentioned above, other FDA-approved anti-angiogenic drugs exist, such as Sunitinib, Sorafenib or Axitinib, among others [36,37].

Although anti-angiogenic drugs have shown an increase in patients’ overall survival (OS), the treatment is usually followed by relapse in tumor angiogenesis and growth [38], which is associated with the use of discontinued therapies [21]. In addition, these patients can also present resistance to the treatment. Resistance to anti-VEGF therapy can explain the high variability found in the clinic using anti-VEGF/VEGFR approaches. Resistance can be classified into intrinsic resistance and acquired resistance. Intrinsic resistance involves tumors failing from the beginning of the treatment: they do not actually respond to this therapy. Acquired resistance happens when tumors initially respond but they result in relapse during treatment [39]. Moreover, the tumor vasculature is heterogeneous and some vessels can respond differently to angiogenic treatment from other vessels. It is accepted now that “new” tumor blood vessels respond better to VEGF-targeted therapies than the more “mature” vessels [40,41]. It is believed that, during maturation, blood vessels lose their sensitivity to anti-VEGF therapy [40]. This is the reason for the use of combinatory therapy of PDGFRβ —involved in pericyte recruitment and then vessel maturation— with anti-VEGF therapy. TKIs are very relevant clinically because they target both VEGF and PDGF pathways [28].

However, even when using combinatorial therapy against VEGF and PDGF, there are numerous alternative pro-angiogenic signaling pathways that can be activated when the VEGF signaling pathway is blocked: angiopoietins, epidermal growth factor (EGF), Delta–Notch pathway, fibroblast growth factor (FGF), hepatocyte growth factor (HGF), placental growth factor (PIGF) or TGF-β/BMPs [42,43], whose inhibition could overcome the limitation of anti-VEGF therapy in either monotherapy or in combination.

Despite the benefits of the use of anti-angiogenic therapy during the last 30 years, this approach has demonstrated several limitations, especially in some types of cancer such as breast or pancreatic cancer [22,28]. As mentioned before, there are different types of resistance to anti-angiogenic therapy, intrinsic or acquired, and numerous limitations to overcome [39]. Moreover, pre-clinical studies have demonstrated that anti-angiogenic therapy can favor tumor hypoxia development and drive to metastasis incidence [44]. In experimental models of metastases, the anti-angiogenic drug sunitinib increased the incidence of metastases after short term treatment [45]. Moreover, in the RIP-Tag2 model, inhibition of VEGFR2 with the monoclonal antibody DC101 or treatment with sunitinib promoted progression, invasiveness and higher incidence of liver metastases [46]. On the other hand, angiogenesis inhibitors are responsible for reduced delivery of chemotherapeutic agents due to a reduction in blood vessel density [21]. Consequently, it is essential to develop different strategies to overcome these limitations.

### 2.2. Vascular Normalization

As mentioned above, targeting the VEGF/VEGFR2 axis could lead to hypoxia due to the excessive pruning of the tumor vasculature that may reduce the delivery of anti-cancer agents, affecting altogether to the efficacy of the treatment. Clinical observations and experimental validation demonstrated that VEGFR2 blockade creates a “normalization window” in which tumor oxygenation and pericyte coverage are increased. This “normalization window” provides a favorable tumor microenvironment that enhances radiation response [47]. Thus, the concept of vascular normalization was then established and it emerged as a promising therapeutic approach [48,49]. This concept is based on the fact that lower doses of anti-angiogenic effects can “normalize” the tumor vasculature, increasing pericyte coverage, which improves vessel stabilization, and reducing vascular permeability and leakiness [50,51]. Normalization of tumor vasculature has been observed using numerous preclinical models [52,53]. Vascular normalization reduces hypoxia and increases the delivery and efficacy of cytotoxic agents, important limitations observed in anti-angiogenic therapy. As tumor vasculature creates an immunosuppressive microenvironment, this normalized vasculature provides a favorable tumor microenvironment [54]. Apart from the VEGF/VEGFR pathway, several strategies have been tested to normalize tumor vasculature. For example, administration of ABTAA (Angiopoietin 2-Binding and Tie2 activating antibody) normalizes tumor blood vessels and polarizes macrophages in orthotopic glioma. Moreover, this treatment reduces tumor growth and metastasis incidence in Lewis lung carcinoma (LLC) tumors [53].

The favorable tumor microenvironment observed during vascular normalization has encouraged scientists to normalize blood vessels to enhance immunotherapy efficacy. By using orthotopic and spontaneous models of breast cancer, the combination of Angiopoietin-2 (Ang2) and VEGF inhibition normalized tumor blood vessels and activated infiltrating CD8^+^ T cells. The antitumor effects of VEGF and Ang2 inhibition were enhanced using PD-1 inhibition [55]. Similar results were observed in glioblastoma models, in which a high abundance of CD8^+^ cells were observed in tumors treated with inhibition of VEGF, Ang2 and PD-1 [56].

Despite the evidence mentioned above, strategies based on vascular normalization are difficult to apply in a clinical setting. On the one hand, it is necessary to develop effective ways to find the correct dose of each anti-angiogenic agent to achieve the desired effect. On the other hand, these effects appear to be temporary, and it is necessary to increase this time window in order to achieve its potential utility in clinical settings for a variety of solid cancer types [22].

### 2.3. Vascular Promotion Therapy

In 2009, Reynolds et al. demonstrated that the administration at a low dose of αvβ3 integrin and αvβ5 integrin inhibitors, Cilengitide and S36578, led to increased tumor growth and tumor angiogenesis. This increase was due to an enhancement of VEGFR2 levels [57].

This new and unexpected effect of cilengitide, which is used as an anti-angiogenic at a high dose, was explored to improve drug delivery in solid tumors. Thus, the administration of low dose cilengitide (ldCil) in combination with gemcitabine and verapamil, a calcium channel blocker which acts as a vasodilator, led to an increase in blood flow that reduced tumor hypoxia and increased drug delivery. This triple combination reduced tumor growth in LLC subcutaneous tumors, A549 experimental metastases, DT6066 orthotopic pancreatic tumors and also KPC tumors, increasing survival and reducing adverse side effects [19,44]. This strategy was named “vascular promotion” and aimed to overcome the limitations of anti-angiogenic therapy (poor drug delivery and hypoxia) and vascular normalization (which causes a very narrow window of opportunity for therapy) [22].

A similar strategy using ldCil was performed with liposomes loaded with doxorubicin and carrying ldCil (MC-T-DOX). Ex vivo assays demonstrated a pro-angiogenic effect of MC-T-DOX. In vivo mice bearing BxPC pancreatic tumors show reduced tumor growth [58].

Eribulin mesilate (eribulin, E7389) is an analog of the marine compound halichondrin B, which is described as an inhibitor of microtubule dynamics. Apart from its strong anti-tumor effects, it has been shown to exert a pro-vascular phenotype in tumors, which makes it a powerful tool for vascular promotion therapy. In the MDA-MB-231 and MX-1 tumor xenografts models, eribulin increases blood vessel density, reduces hypoxia and controls tumor growth when it is administered in combination with the chemotherapeutic agent capecitabine [59]. The effect of eribulin is clinically relevant, as it has been demonstrated in clinical trials. Patients with metastatic breast cancer who respond after eribulin treatment show an improved tumor microenvironment, as demonstrated by lower Foxp3^+^ cells, higher levels of CD8^+^ cells and lower levels of PD-L1 [60]. Similar effects were found in metastatic breast cancer patients treated with paclitaxel and eribulin, in which patients who respond to this combination show lower levels of hypoxia and higher levels of epithelial markers such as E-cadherin [61], suggesting that the promotion of a favorable microenvironment by eribulin is associated with a lower hypoxia and lower epithelial-to-mesenchymal transition (EMT) program. More recently, the use of eribulin as a vascular-promoting effect to treat tumors has been described in xenografts models of clear cell sarcoma, in which tumor growth was controlled via vascular remodeling [62].

Apart from ldCil and eribulin, other pathways have been explored as tools for vascular promotion therapy. Lysophosphatidic acid (LPA) has been demonstrated to promote a fine capillary network in experimental models of Lewis lung carcinoma (LLC). Its effect as a pro-vascular agent is due to the promotion of endothelial cell contacts via localization of VE-cadherin. This effect reduces tumor hypoxia and improves drug delivery. This was functionally demonstrated when LPA was administered in combination with 5-fluororacil or oxaliplatin and this combination reduced tumor growth [63].

## 3. BMP9–ALK1 Signaling Axis

Bone morphogenetic protein 9 (BMP9) is a cytokine that belongs to the highly conserved transforming growth factor (TGF-β) superfamily of proteins. This superfamily includes a massive pool of growth factors such as the TGF-β subfamily (with five isoforms: TGF-β1 to 5), the bone morphogenetic protein (BMP) subfamily, the activins and inhibins subfamilies, the nodal group, the growth differentiation factors (GDF) subfamily and the anti-Müllerian hormone, among others [64,65].

All the ligands from the TGF-β superfamily bind to a complex of dimers composed of type I receptors (ALK1-ALK7, activin receptor-like kinase 1 to 7) and dimers of type II receptors (BMPRII or ActRII), leading to the activation of Smad dependent signaling cascades [66,67,68]. The main difference between BMP and TGF-β signaling is that the Smad signaling pathway is activated through ALK receptors. It is well accepted that BMP signaling transduces signals through the Smad1, Smad5 and Smad8 pathways, whereas TGF-β does it through the Smad2 and Smad3 pathways. Both Smad1/5/8 and Smad2/3 share co-Smad4 to transduce signals into the nucleus, and this leads to a lateral antagonism between both pathways [69,70,71].

### 3.1. Bone Morphogenetic Protein 9 (BMP9)

BMP9 is part of the BMPs, the largest subgroup of signaling molecules in the TGF-β superfamily. They were first discovered due to their ability to induce bone and cartilage formation [72], but they also participate in embryonic development, adult tissue homeostasis, control of stem cells, fracture repair, and cellular differentiation [64,73,74,75]. BMP9 is encoded by the *GDF2* gene (chromosome 10q11) in humans. Pathogenic mutations in this gene cause a subtype of a vascular rare disease called hereditary hemorrhagic telangiectasia (HHT), although the total contribution of BMP9 mutations is estimated to be <1% [76].

BMP9 was first identified as an autocrine and paracrine mediator, expressed predominantly in the liver, that induces proliferation in cultured liver cells [77]. It has been described as both a pro-angiogenic [78] and an anti-angiogenic factor [79,80] through numerous in vitro and in vivo experiments. This dual role is dependent on many factors: heterogeneity of blood vessels, and presence of other receptors and ligands, among others [81,82]. BMP9 has been shown to be a vascular quiescence factor, inhibiting endothelial cell migration and proliferation, and it has also been identified as a hematopoietic, hepatogenic, osteogenic and chondrogenic factor [83,84].

This cytokine acts as one of the principal ligands of two specific endothelial cell surface receptors: endoglin and the activin receptor-like kinase 1 (ALK1).

### 3.2. Endoglin (CD105)

Endoglin (CD105) is a type I membrane glycoprotein, encoded by the *ENG* gene, that acts as a co-receptor of the TGF-β superfamily and is mainly expressed in ECs [85]. Endoglin contains a long extracellular domain, a transmembrane domain and a short intracellular tail, which allows endoglin to act as a co-receptor, since it requires the presence of other receptors to induce signaling [85]. There are two isoforms that differ in the length of the intracellular domains that are produced by alternative splicing: long endoglin (L-Endoglin), the majority isoform, and short endoglin (S-Endoglin) [86].

### 3.3. Activin Receptor-Like Kinase 1 (ALK1)

ALK1 is a type I cell surface receptor for the TGF-β/BMP superfamily that interacts with different ligands such as TGF-β1 or BMP9, among others. This receptor is mostly expressed in endothelial cells and participates in the regulation of angiogenesis, wound healing, tissue repair and tumor angiogenesis [75,87,88]. ALK1 is encoded by the *ACVRL1* gene (chromosome 12q13) in humans and, similar to BMP9, pathogenic mutations in this gene cause a subtype of HHT, type 2 HHT. Mutations in this gene and endoglin are the cause of approximately 85% of cases of HHT [76].

**Figure 2:**
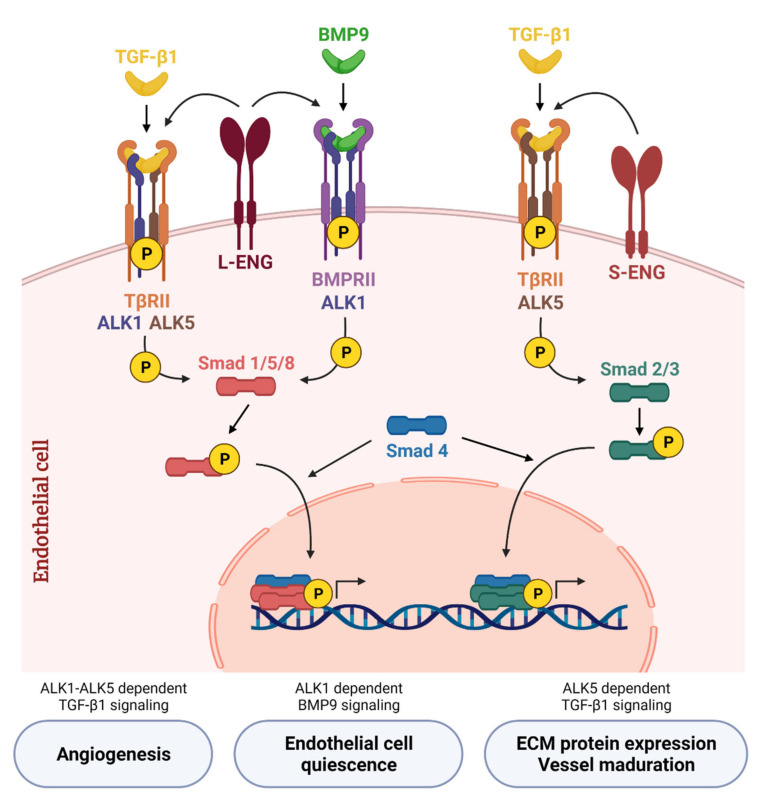
**Canonical TGF-β/BMP signaling network in endothelial cells**. TGF-β1 binds to the transforming growth factor-β receptor type II (TβRII) that phosphorylates the type I receptor (TβRI). In endothelial cells, TGF-β1 induces Smad2/3 phosphorylation through TβRI activin receptor-like kinase 5 (ALK5) and is potentiated by S-Endoglin (S-ENG) (**right part of the scheme**). On the other hand, TGF-β1 induces Smad1/5/8 phosphorylation through TβRI activin receptor-like kinase 1 (ALK1)—only in the presence of ALK5—and is potentiated by L-Endoglin (L-ENG) (**left part**). BMP9, the high affinity ligand for ALK1, transduces signals through ALK1 and bone morphogenetic protein receptor II (BMPRII). BMP9 binds to this receptor complex and activates the Smad1/5/8 signaling pathway, which regulates the balance between quiescent or activated endothelium depending on the cellular context. L-ENG is part of this receptor complex and potentiates BMP9-induced effects (**middle part**). Created with BioRender.com, accessed on 17 October 2021.

ALK1 receptor was discovered as an important regulator of the cardiovascular system when ALK1 knock-out mice were found to die during embryogenic development. It is an important regulator of the angiogenic process via the TGF-β1 pathway [89]. Goumans et al. demonstrated that TGF-β1 can activate two type I receptors: ALK1 and ALK5. Cell signaling through ALK1 activates Smad1/5 phosphorylation and promotes the expression of Inhibitor of differentiation 1 (Id1), while cell signaling through ALK5 activates Smad2 phosphorylation and promotes the expression of Plasminogen activator inhibitor 1 (PAI-1). Id1 is involved in endothelial proliferation and migration while PAI-1 is involved in vessel maturation. Thus, both ALK1 and ALK5 regulate the endothelial balance between quiescent or activated: when TGF-β1 activates the Smad1/5 pathway through ALK1, the activation phase of the angiogenesis is promoted by EC proliferation and migration, but when TGF-β1 activates Smad2/3 through ALK5, the resolution phase of angiogenesis is promoted [90]. Moreover, it was described that, at a molecular level, the cellular signaling by Smad1/5 induced through ALK1 is a lateral antagonist of the ALK5-Smad2/3 pathway receptor. However, more interestingly, it was demonstrated that TGF-β1 induces signals through ALK1-Smad1/5 only in the presence of ALK5. Thus, a receptor heterocomplex ALK1/ALK5 is necessary for TGF-β1 effects in endothelial cells [69,91]. The co-receptor endoglin (CD105) was shown to be essential to promote TGF-β1 signaling through the ALK1-Smad1/5/8 pathway in endothelial cells. Ectopic expression of endoglin potentiates ALK1-Smad1/5/8 signals and its consequent activation of endothelial proliferation and migration. Moreover, endoglin blocks TGF-β1 cell signaling through the ALK5 receptor, and thus inhibits the quiescent state of the endothelium [70,71,92]. This role of endoglin inhibiting ALK5-Smad2/3 pathway, and thus inhibiting extracellular matrix deposition, was reported in L6E9 rat myoblasts [93], although one year later two distinct roles of L-Endoglin and S-Endoglin were described in depth. While L-Endoglin promotes TGF-β1 signaling through ALK1-Smad1/5/8, S-Endoglin promotes TGF-β1 signaling through ALK5-Smad2/3 and increases extracellular matrix synthesis (Figure 2) [86].

### 3.4. BMP9–ALK1 Signaling

The crystal structure of BMP9 and its potential interactions with ALK1 were determined in 2005 [94]. Two years later, David et al. identified BMP9 and BMP10 as high affinity ligands for ALK1 in human dermal microvascular endothelial cells [79].

In detail, canonical BMP9–ALK1 signaling starts when BMP9 binds with high affinity to ALK1 followed by the recruitment of BMPRII, and the auxiliary receptor L-endoglin. These are cell surface receptors with serine/threonine kinase activity, hence, the BMPRII activates ALK1 by phosphorylation [73,95]. The formation of this complex subsequently triggers the phosphorylation of the regulatory Smads proteins (R-Smad), Smad1/5/8. As explained before, these activated Smads form a heteromeric complex with the Co-Smad, Smad4, and enters into the nucleus, which promotes the expression of different target genes [73,74,75,78] (Figure 2). The main effector pathway of BMP9 signaling is Smad1/5/8, although it can vary depending on the cell type and cellular context. BMP9 transduces signals through Smad1/5/8, as it has been described in numerous studies on endothelial cells [79,96,97,98,99,100,101,102]. In the hepatocellular carcinoma cell line HepG2, BMP9 induces phospho-Smad1/5/8 phosphorylation leading to increased survival [103]. In mouse embryo fibroblasts it was found that BMP9 activates Smad1/5/8 through both ALK1 and ALK5 receptors, leading to an increase in extracellular matrix proteins collagen I, fibronectin and connective tissue growth factor (CTGF) [75]. Although sometimes transient, BMP9 has also been shown to activate Smad2/3 pathways in human endothelial cells [104], glioblastoma cell lines [105] or mouse embryo fibroblasts [75]. Currently, new mechanisms of the regulation of ALK1 activity are being described. Fu et al., have demonstrated that ALK1 activity and endothelial balance can be regulated by ubiquitination [106]. In this way, OTU deubiquitinase with linear linkage specificity (OTULIN) and the linear ubiquitin chain assembly complex (LUBAC) inhibit ALK1 ubiquitination, thus inducing its downstream Smad1/5 phosphorylation [107].

Besides the Smad-dependent signaling pathway, there is also a non-canonical signaling via mitogen-activated protein kinase (MAPKs), phosphatidylinositol 3-kinase/protein kinase B (PI3K/Akt), AMP-activated kinase (AMPK), small GTPase Ras-ERK pathways, c-Jun N-terminal kinase (JNK), and others [64,65,74]. The activation of these pathways has been demonstrated by numerous cell types. In HepG2 cells, BMP9 activates Akt and p38 pathways inducing proliferative responses [108]. It has also been described that BMP9 induces the activation of p38 in oval cells, promoting cell death [109]. In glioblastoma cell lines, BMP9 activates PI3K, p38, Akt and Erk1/2 to promote invasiveness [105]. Nevertheless, not much is known about the activation of these non-canonical pathways, and furthermore most of the data have been obtained from mesenchymal progenitor cells.

### 3.5. BMP9–ALK1 Axis and Endoglin

As mentioned previously, L-endoglin promotes signaling through ALK1 and Smad1/5/8 and S-endoglin through ALK5 and Smad2/3 (Figure 2) [86]. Since the first functional studies that describe the molecular effects of endoglin, it was demonstrated that endoglin overexpression increased BMP9-induced response [79].

There is also a soluble form of endoglin (sEng), which is released by the action of MMP-14 on the extracellular domain and is present in the serum of patients with cancer and other diseases [110]. sEng has anti-angiogenic properties—in fact, it has been described acting as a ligand trap for BMP9, preventing type II receptor binding and thus inhibiting BMP9 signaling activity [111]. The involvement of endoglin in the regulation of angiogenesis was used in endoglin heterozygous mice (*Eng*^+/−^) and their role in tumor growth was also confirmed using these mice [112]. The important role of endoglin as part of this receptor complex and its capability to modulate its cellular signaling and biological effects show it as an interesting therapeutic target, which we will detail later.

### 3.6. BMP10 and BMP9 in the Vascular Context

BMP9 and BMP10 were identified at the same time as ligands of ALK1. Both proteins have been demonstrated to behave as important vascular homeostasis regulators [79]. Traditionally it was believed that both proteins were redundant, but current studies using neutralizing antibodies or deficient mice are showing different functions for these proteins [113]. BMP9 is expressed in the liver, with this expression being restricted to hepatic stellate cells (HSCs) [77,114,115,116]. In contrast, BMP10 is expressed in the heart and restricted to the right atrium [117,118,119]. Both BMP9 and BMP10 are circulating factors displaying activity in the serum [120]. Circulating BMP9 and BMP10 are active in a BMP9–BMP10 heterodimeric form [114] and bind to ALK1 receptors with picomolar affinities [94]. BMP9 also binds to ALK2 but with much less affinity, while BMP10 does not bind to ALK2 but has been proposed to bind to ALK3 or ALK6 [121]. Moreover, regarding type II receptors, it seems that BMP9 shows differences in binding ActRIIB, BMPRII and ActRIIA [122]. However, both BMP9 and BMP10 bind to the co-receptor endoglin (CD105), which, as mentioned before, potentiates the signaling activity [123]. Studies using *Bmp9* knockout mice (*Bmp9*-KO) and *Bmp10*-KO have evidenced different physiological roles for both cytokines. Firstly, *Bmp9*-KO mice are viable and fertile, whereas *Bmp10*-KO die during embryonic development [124,125]. As mentioned, *Bmp10-*KO mice develop alterations in heart development, which are not observed in *Bmp9*-KO mice. These *Bmp9*-KO show enlarged lymphatic vessel and valves due to a higher expression of LYVE-1, a protein involved in the lymphatic vessel maturation of BMP9–ALK1 [126]. The same research team demonstrated later that *Bmp9*-KO mice presented impaired closure of the ductus arteriosus in neonates and this effect was higher after treatment with anti-BMP10 neutralizing antibody [127]. These results confirm that BMP9 and BMP10 have complementary roles and not redundancy. As we will review in the following sections, BMP10 seems to have different effects on tumor angiogenesis than BMP9 and BMP10 may explain the discrepancies observed between ALK1 targeting and direct BMP9 inhibition.

## 4. BMP9–ALK1 as Modulators of Tumor Angiogenesis: In the Focus for Cancer Treatment

### 4.1. Targeting BMP9–ALK1 Axis: A Good Anti-Angiogenic Strategy?

The angiogenic effect of ALK1 was demonstrated by using genetic deletion of ALK1 (ALK1 heterozygous mice, *Acvrl1*^+/−^) in a mouse model of tumor angiogenesis, the RIP1-TAg2 model of insulinomas [128], RIP1-TAg2; *Acvrl1*^+/−^ mice developed lower tumor angiogenesis and lower tumor burden. This effect was also reproduced by pharmacologic inhibition of ALK1 [129]. Similarly, pharmacologic inhibition of ALK1 reduced tumor growth and tumor angiogenesis in the MMTV-PyMT model (mouse model of breast cancer metastasis) and decreased the incidence of lung metastases [130]. Surprisingly, BMP9 deficiency increased branching and associated liver metastases, suggesting different roles of BMP9 and ALK1 [131].

Ex vivo models of angiogenesis demonstrated that the pro-angiogenic cytokines VEGF and FGF stimulated ALK1-Smad1/5/8 and this results in an induction of cell spread and tubulogenesis. Using a xenograft model of melanoma resistant to anti-VEGF, ALK1 inhibition delayed tumor growth and disturbed the maturation of blood vessels. This suggests the existence of an independent mechanism of ALK1 in tumor angiogenesis and the usefulness of using anti-ALK1 in combination with anti-VEGF in those tumors not sensitive to this therapy [132].

The evidence of the involvement of ALK1 in vascular development and angiogenesis in vitro and in vivo soon stimulated the scientific community to start clinical trials to develop molecules that target ALK1 to inhibit tumor angiogenesis and tumor progression, some of them mentioned before. The first molecule was a soluble chimeric protein called ALK1-Fc (Dalantercep), which has been shown to inhibit the proangiogenic effects of ALK1 in vitro. At a molecular level, it blocks BMP9 and BMP10, binding and downstream Smad1 pathway activation. Treatment with ALK1-Fc led to a reduction in VEGF and FGF-induced sprouting [133]. Additionally, in vitro studies with PF-03446962, an anti-human ALK1 antibody, reduced BMP9-induced Smad1 phosphorylation and blocked VEGF-induced sprouting [134].

In the RIP1-TAg2 multistep mouse model of tumor angiogenesis, it was shown that the inhibition of ALK1 activity with the ALK1-Fc fusion RAP-041 retarded tumor growth. At a molecular level, RAP-041 neutralized BMP9 activity and inhibited angiogenic sprouting in vitro and in vivo [129].

The anti-angiogenic effect of the pharmacologic inhibition of ALK1 with ALK1-Fc was also demonstrated to have an impact on metastasis incidence. Thus, treatment with RAP-041 was shown to decrease vessel area in the RIP1-Tag2 mouse model, and this was associated with a lower incidence of liver metastases. Similar results were confirmed in breast primary tumors using the MMTV-PyMT model, in which ALK1-Fc treatment reduced tumor volume and vessel area in the primary tumor, and it also reduced the number of lung metastases [130]. In xenografts models of head and neck cancer and in murine models of melanoma, treatment with ALK1-Fc led to a reduction in blood vessel area without affecting tumor growth when used in combination with chemotherapy [135]. The use of ALK1-Fc as an anti-angiogenic molecule has been demonstrated when administrated in monotherapy and in combination with chemotherapy. As previously indicated, ALK1-Fc in combination with docetaxel was shown to control primary tumor growth in the MMTV-PyMT model, decrease blood vessel density in the primary tumor growth and reduce the metastatic colonization in the lungs [130]. On the other hand, the administration of ALK1-Fc controlled tumor growth when it was administered with cisplatin or doxorubicin in the orthotopic breast cancer model using KEP1-11 cells [135].

**Figure 3:**
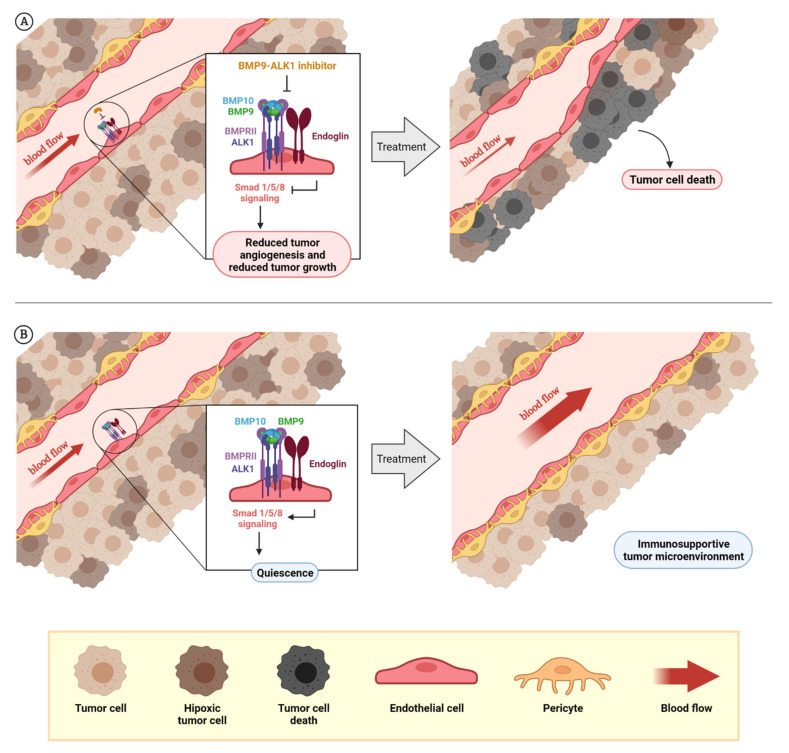
**Rationale for the design of BMP9–ALK1-based therapies**. The dual role of BMP9–ALK1 in endothelial balance can be used for different therapeutic approaches: the inhibition of tumor blood vessels in which ALK1 behaves as a pro-angiogenic receptor (BMP9 triggers its signaling through Smad1/5/8 and promotes the activation of angiogenesis) that can be used to reduce tumor angiogenesis and tumor growth, as has been demonstrated with molecules such as ALK1-Fc or PF-03446962. Additionally, as endoglin expression in endothelial cells potentiates BMP9-induced effects, blocking endoglin with molecules such as TRC105 can cause the same effects (**A**). On the other hand, considering blood vessels where the activity of the circulating factor BMP9 induces vessel quiescence through ALK1 receptor, the potentiation or mimicry of these effects can be used for vessel normalization with therapeutic purposes. Here is the opportunity to explore the effects on vascular normalization and creating a favorable tumor microenvironment of recombinant BMP9 administration or analogues (**B**). The composition of BMPRI (ALK1 or ALK5) and BMPRII receptor complexes and the levels of the ligands BMP9 and BMP10 seems to be very important to determine when ALK1 receptor induces the promotion of angiogenesis or vessel maturation. At the same time, the role of other factors such as TGF-β1 or sEng, which can block BMP9 binding, needs to be further studied. Created by BioRender.com, accessed on 17 October 2021.

All these studies and current clinical trials evidence the potential use of ALK1 as an anti-angiogenic drug [136,137] (Figure 3A), some of them with potential use in monotherapy or in combination with chemotherapies and others, perhaps in combination with other anti-angiogenic drugs. For example, this year two macrocyclic small molecular compounds (OD16 and OD19) have been described to inhibit Smad1/5 signaling induced by BMP9 through ALK1/2 receptors. They have shown higher selectivity than other ALK1/2 inhibitors previously used, like LDN-193189. Both molecules have proved in vitro anti-angiogenic properties that are induced by BMP9 and VEGF [138].

#### Strategies Based on Endoglin Inhibition

As mentioned before, endoglin (CD105) is essential for the BMP9–ALK1 signaling pathway and plays an important role in tumor biology and tumor angiogenesis [139]. Therefore, the concept of endoglin inhibition as an anti-angiogenic and anti-tumor strategy has been used to develop anti-endoglin therapies, such as TRC105, a chimeric IgG1 monoclonal antibody developed by TRACON Pharmaceuticals [140,141]. This antibody, Carotuximab (TRC105), binds to the orphan domain of the extracellular domain of endoglin in proliferating endothelial cells, preventing BMP9 binding and Smad1/5/8 phosphorylation. Thus, it maintains quiescence and inhibits angiogenesis in vitro and in preclinical cancer models [140,142]. This involves BMP9 competitive inhibition as a mechanism of action of anti-endoglin antibodies [140]. As well as competing with BMP-9, TRC105 induces the release of sEng via MMP-14, which can act as a ligand trap to inhibit angiogenesis [142].

TRC105 has also shown immune-dependent mechanisms since it appears to target immunosuppressive components in the TME like regulatory T cells (Tregs) [143]. Therefore, targeting endoglin with TRC105 can affect the regulation of the immune components of TME.

In the clinic, the results obtained with TRC105 have been promising in different types of cancers, especially when combined with other antiangiogenic drugs. TRC105 has been tested in different clinical trials [142]. For example, it has been tested in phase I and II clinical trials for prostate cancer [144], hepatocarcinoma [145] and other types of solid tumors [146], and in phase III in patients with angiosarcoma, although so far no clinical benefits have been observed [147].

The fact that endoglin, as another member of the BMP9–ALK1 receptor complex, can be targetable, offers additional opportunities to inhibit BMP9–ALK1-induced angiogenesis: BMP9–ALK1 can be targeted directly or indirectly via endoglin inhibition.

In summary, direct inhibition of BMP9–ALK1 via endoglin targeting aroused interest and numerous clinical trials have been performed over the last 20 years (Table 1) based on the pro-angiogenic properties of the BMP9–ALK1-endoglin axis (Table 1).

### 4.2. Improving Anti-Angiogenesis Limitations: Is the BMP9–ALK1-Induced Quiescence an Interesting Tool for Vascular Normalization?

Although the normalization of tumor vasculature and its anti-tumor effect has been demonstrated to target multiple pathways such as VEGF or Ang2, there is little evidence about the consequences of targeting BMP9–ALK1 in vessel normalization and its anti-tumor effect.

Hu-Lowe et al. demonstrated an interesting result when using ALK1 inhibition. In the M24met/R xenograft model, inhibition of ALK1 led to an increase in blood vessel density and a disruption in the adhesion between endothelial cells and pericytes [132]. This study actually demonstrated the clear effect of ALK1 on the maturation and normalization of tumor blood vessels. However, to understand this, we need to refer to previous studies performed in vitro and in non-tumor contexts.

Contrary to previous studies published in the early years of the 21st Century, Lamouille et al. reported that ALK1 was involved in the maturation phase of angiogenesis using in vitro studies with human microvascular endothelial cells and human umbilical vein endothelial cells (HUVEC), where they described the inhibitor role of ALK1 in endothelial cell migration [148]. At the molecular level, this mechanism was regulated by ERK and JNK pathways [83]. As mentioned before, this research identified first both BMP9 and BMP10 as high affinity ligands for ALK1 receptors in endothelial cells. BMP9 induced the phosphorylation of the phospho-Smad1/5/8 pathway, and both cytokines induced EC migration, reproducing the effects of ALK1 overexpression [149]. After that, this research group identified BMP9 from serum and demonstrated a quiescent state-maintaining role of this cytokine in the chick chorioallantoic membrane (CAM) assay [84]. This important role of BMP9 in vascular quiescence was also proved in postnatal retinal development. It was shown that anti-BMP9 treatment increases the vascularization of the retina, suggesting an important role of BMP9 in retinal vascular remodeling [124]. In parallel with the studies performed by Dr. Bailly’s team, Sharpfenecker et al. demonstrated that BMP9 inhibits EC proliferation and migration and VEGF-induced sprouting through the Smad1 pathway [80]. In concordance with these results, Larrivée et al. described that BMP9 induced the expression of genes such as HES1, HEY1 and HEY2, which, together with Notch signaling, reduced the VEGF-response, contributing to endothelial stability and quiescence [150]. Kerr et al. characterized a small molecule kinase inhibitor (K02288), which inhibited BMP9-induced Smad1/5/8 phosphorylation. Treatment with K02288 in endothelial spheroids resulted in a hypersprouting phenotype, showing similar effects to ALK1-Fc phenotype [151]. Assessing the possible role of BMP9–ALK1 in the tumor vasculature, it was shown in the RIP-Tag2^+^ pancreatic insulinoma model that genetic ablation of *Bmp9* led to an increase in branching and increased metastasis, confirming the role of BMP9 in vessel quiescence observed previously. However, genetic ablation of *Alk1* decreased vasculature and metastases, suggesting that BMP9 and ALK1 do not share the same functions, or that any other ligand, such as BMP10, can have a different role to BMP9. This exhibits the complexity of the BMP9–BMP10/ALK1 axis [131].

Using an orthotopic model of breast cancer with E0771 cells, Ouarné et al. demonstrated different roles for both BMP9 and BMP10 proteins in vascular phenotypes. Using mice lacking BMP9 (*Gdf2*^−/−^), they observed an increase in primary tumor growth and a decrease in vessel perfusion and vessel maturation, with an associated increased spontaneous metastasis, confirmed by the number of lesions, metastatic area, and the size of the lesions. This suggests the important role of BMP9 in vessel normalization and its effect in the tumor microenvironment. Interestingly, BMP10 deletion does not have any effect and mice lacking both factors (*Gdf2*^−/−^, *Bmp10^flox/flox^*) do not exhibit improved effects of BMP9, demonstrating the individual role of the ALK1 ligand BMP9 in vascular normalization [152].

Further to previous studies, Viallard et al. demonstrated that the overexpression of BMP9 in LLC tumors promoted the normalization of the vasculature. LLC-overexpressing BMP9 show normalized and increased blood vessel density, pericyte coverage and perfusion. This is due to the ability of BMP9 to stabilize endothelial junctions. Moreover, BMP9-induced vascular normalization led to a reduction in tumor hypoxia, with a consequent increase in T cells (CD4^+^ and CD8^+^) [153]. The observations made by these authors about the effect of BMP9 in the tumor microenvironment and immune cell infiltration are very interesting. They observed that BMP9-expressing LLC tumors show increased immune cell infiltration. Looking at RNA signatures, they observed an increase in leukocyte-endothelial adhesion molecules such as KLF4, CCR2, VCAM1, CCL2, TNF, CXCL12 or NFAT. Moreover, they demonstrate that the increased immune cell infiltration is mainly due to the effect of BMP9 in vascular cells and not due to a direct effect of BMP9 in T cell activation, since T cell activation and proliferation were not increased upon in vitro stimulation with BMP9 [11]. These results are in concordance with other studies that describe the role of BMP9 in immune cell infiltration in non-tumoral tissues [154].

The usefulness of BMP9 as a vessel-normalizing factor is very interesting and relevant to the clinic. Further studies that deliver recombinant BMP9 directly to the tumor, thus normalizing tumor vasculature and creating a favorable tumor microenvironment, will show us if this approach can offer benefits to patients (Figure 3B).

As exposed above, the main limitation of vascular normalization is that most of the vascular normalizing strategies are temporary, and this temporary window should be opened for cancer treatment in clinical settings [22]. Controlling this “normalization window” by administering BMP9 is very important as the molecular landscape in the blood vessels can vary and different BMP9-induced effects can occur, especially considering the dual effect of this pathway. This is especially relevant if we want to combine vascular normalizing molecules, such as BMP9, which promote an immunostimulatory tumor microenvironment and immunotherapies.

### 4.3. Vascular Promotion Therapy: Is the Promotion of the Pro-Angiogenic Effect of BMP9–ALK1 a New Tool for Vascular Promotion?

The dual effect of the BMP9–ALK1 pathway is very interesting to explore in vascular promotion therapy. First, the inhibition of this pathway in the contexts where BMP9–ALK1 is promoting quiescence can be very interesting to awaken blood vessels. Mimicking the ALK1-proangiogenic effects that can be induced by administration of recombinant BMP10 or BMP9 in the tumors with blood vessels where ALK1 behaves as a pro-angiogenic receptor. On the other hand, as demonstrated previously, in some contexts, the inhibition of ALK1 can disrupt vessel normalization [132] and this activation of the angiogenic phase of tumor blood vessels can benefit “vascular promotion” therapies. However, it is necessary to mention that currently there are no known perspective biomarkers to predict the pro-angiogenic or anti-angiogenic effect of BMP9–ALK1 therapeutic modulation. In this direction, finding a correlation between the presence of TβRII, BMPRII or other ALK receptors can be a starting point for discovering this predictive and prognostic value.

## 5. BMP9 and Its Effects beyond the Tumor Vasculature: Is There Another Chance?

### 5.1. BMP9 Induces Direct Effects on Tumor Cells

**Figure 4:**
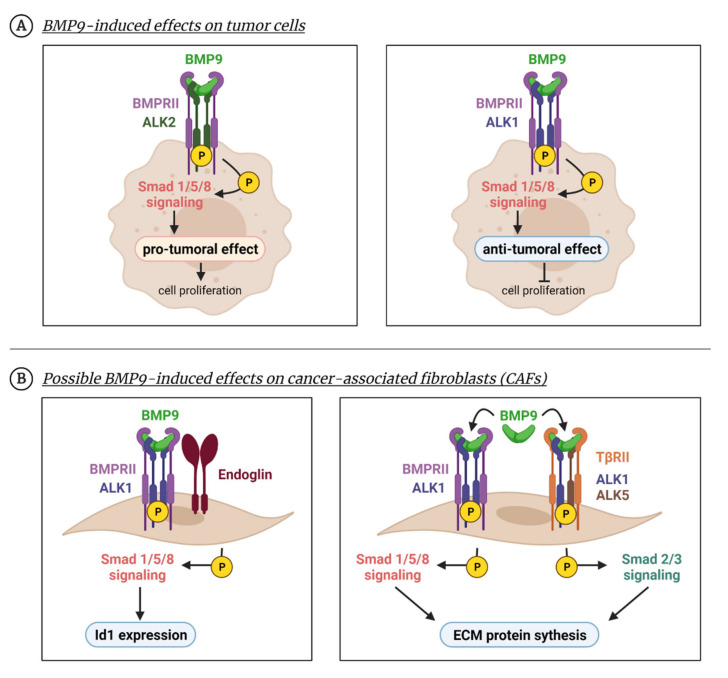
**BMP9 effects beyond the endothelium. BMP9 can produce cellular effects in non-endothelial cells as numerous studies have shown in tumor cells or fibroblasts**. (**A**) BMP9 enhances tumor cell survival in several cancer cell types due to the activation of the Smad1/5/8 pathway through ALK2 receptor (**left panel**). Apart from Smad1/5/8, other non-canonical pathways such as p38 are activated. In different tumoral cell lines, BMP9 induces an anti-proliferative effect by activating the Smad1/5 pathway through the ALK1 receptor (**right panel**). (**B**) BMP9 can induce responses in fibroblast-like cells, such as CAFs, in which BMP9 promotes Id1 expression through the ALK1-Smad1/5/8 pathway (**left panel**). In MEFs, BMP9 induces a profibrotic program through both Smad1/5/8 and Smad2/3 pathways (**right panel**). Created with BioRender.com, accessed on 17 October 2021.

In the previous sections, we have described that ALK1 is not completely restricted to endothelial cells and its ligand BMP9 can produce effects in different cell types. In several cancer cell types, especially in liver cancers, BMP9 acts as a pro-tumoral growth factor. Herrera et al. described in 2009 that ovarian carcinoma cells respond to BMP9 stimulation, as Smad1/5/8 is phosphorylated and Id1 is upregulated after treatment with BMP9. This mechanism was ALK2 and not ALK1 dependent. In these cells, treatment with BMP9 increases cell proliferation (Figure 4A) [155]. Li et al., observed that BMP9 is expressed in hepatocellular carcinoma (HCC) human tissue, and that HCC cell lines HepG2 and HLE respond to the BMP9 activating Smad1/5/8 pathway. Treatment with BMP9 induces the EMT program in these cells, with both ALK1 and ALK2 being necessary for this [156]. Moreover, it was shown that HepG2 produces BMP9 and is involved in its proliferation and survival [103]. For HepG2 proliferation, the Smad1/5/8 pathway and other non-Smad pathways such as p38, are required [108]. Besides, treatment with ALK1-Fc also showed an effect in the tumor cell compartment, since in orthotopic models of prostate cancer the inhibition of ALK1 with ALK1-Fc decreased BMP9-induced signaling, the proliferation of tumor cells and tumor growth. Interestingly, ALK1-Fc increased hypoxia and apoptosis [157]. In renal carcinoma, BMP9 also induced EMT and this effect was reversed by using a neutralizing antibody, BMP9-0093 [158].

However, the effect of BMP9 in cancer cell proliferation is unclear. In contrast with these studies, some others found an inhibitory effect of BMP9–ALK1 on tumor cell proliferation and tumor growth (Figure 4A). In the SK-B3-2 breast cancer cell line, BMP9 reduced tumorigenic properties in vitro such as colony formation or migration and decreased tumor growth in vivo [159]. Moreover, BMP9 inhibited the formation of bone metastases from MDA-MB-21 breast cancer cells [160]. Treatment with a recombinant human BMP9 (MB109) inhibited HCC proliferation in vitro [161].

All these results suggest that the inhibition of BMP9–ALK1 could affect the tumor compartment besides the tumor vasculature. However, the role of ALK1 is perhaps not yet elucidated in the tumor cell compartment, as most of the studies identified ALK2 as the main player of BMP9-induced effects in tumor cells. Thus, numerous studies identifying the affinities and binding properties for this receptor will be essential regarding pharmacokinetic and dose scheduling in future treatments.

### 5.2. BMP9-Induced Effects in Other Components of the Tumor Microenvironment

BMP9 and BMP10 have been described as acting primarily on ECs, where they can promote the secretion of potent vasoactive factors such as endothelin-1 (ET-1) [162]. However, they have been described to act directly on vascular smooth endothelial cells (VSMCs) by binding ALK1. BMP9 and BMP10 are essential for VSMC contractility. ALK1 activation by BMP9 leads to Smad1/5/8 phosphorylation. Interestingly, this effect in these cells takes place in pulmonary blood vessels but not in aortic and coronary arteries. The heterogeneity of this response is related to the differential expression of BMP type I receptors ALK1, ALK2, ALK3 and ALK6 [163]. All these findings have been shown in the context of pulmonary hypertension, but they evidence a possible effect of BMP9–ALK1 in mural cells, supporting cells which stabilize tumor blood vessels, especially pericytes. There is little evidence about the expression of BMP receptors in pericytes and the possible effect of BMP9 in pericyte biology. Future studies addressing the role of BMP9–ALK1 in pericyte recruitment, pericyte proliferation or pericyte–endothelial adhesion would be necessary to understand the effect of BMP9–ALK1 on tumor angiogenesis. On the other hand, pericytes have been described recently to secrete cytokines that directly influence tumor growth [6,164,165]. For these reasons, the study of pericytes is gaining focus in the context of the tumor microenvironment and perhaps BMP9–ALK1 can help us to understand it.

There is very little known about the possible effect of BMP9 in cancer associated fibroblasts (CAFs). CAFs are a major component of the tumor microenvironment that regulate tumor progression, immune cell infiltration, tumor cell metabolism and secrete amounts of extracellular matrix proteins [166,167]. A recent study showed the interesting role of endoglins in CAFs promoting tumor progression and metastasis incidence in colorectal cancer. In these cells, endoglin effects can be induced by BMP9. In vitro studies with CAFs show that BMP9 stimulates Id1 through an endoglin-dependent mechanism in which Smad1/5/8 is phosphorylated (Figure 4B). This effect is blocked by using TRC105 (anti-endoglin) antibody [168]. The role of BMP9 in extracellular matrix protein synthesis was described by our team in 2016 using mouse embryo fibroblasts (MEFs). We demonstrated that BMP9 can stimulate extracellular matrix (ECM) protein synthesis in MEFs by activating both Smad1/5/8 and Smad2/3 pathways in an ALK1–ALK5 dependent mechanism (Figure 4B) [75].

Nevertheless, all these previous observations are only the beginning of more interesting effects and derived opportunities.

## 6. Conclusions and Future Directions

The dual role of BMP9–ALK1 in blood vessel biology has proved to be a very interesting tool for cancer treatment, especially for combination therapy. However, very important challenges arise in the study of BMP9–ALK1 in the modulation of tumor vasculature and its translational value in the clinic.

First, scientists need to know when this pathway behaves as pro- or anti-angiogenic and this different behavior seems to be related to the heterogeneity of tumor blood vessels and the composition of BMPRI (ALK1, ALK2, ALK3 and ALK6) and BMPRII receptors. A second and very interesting challenge is to dissect the effects of both circulating BMP9 and BMP10 proteins in the context of tumor angiogenesis. Moreover, the relationship between both circulating factors and TGF-β1 need to be better elucidated, as TGF-β seems to be an important regulator of the tumor microenvironment [169]. Targeting the BMP9–ALK1 pathway through endoglin inhibition is another tool, and perhaps very useful due to the effects of this co-receptor in immune cells and endothelial–mural cell adhesion, but more interesting is the exploration of the possible therapeutic effects of sEng in BMP9 binding to ALK1 and its downstream activity. However, once again, understanding the heterogeneity of tumor blood vessels and their molecular composition will help to predict the response to inhibition or mimicking of this dual pathway.

On the other hand, the pleiotropy of BMP9 makes all the studies more complex if the effect of targeting this molecule is not restricted to endothelial cells. BMP9-induced effects over other cell types from the tumor microenvironment (like CAFs, pericytes or immune cells) are very important regarding the design of new therapies. At the same time, there is evidence that BMP9 can directly affect tumor cells.

Taken together, BMP9 is a powerful cytokine with numerous pro- or anti-tumorigenic and vascular and non-vascular effects, which makes further studies necessary to elucidate how this potential can be exploited. Many more years of research should shed light on this and reveal whether a novel approach to BMP9–ALK1 targeting in anti-angiogenesis, vascular normalization or vascular promotion therapies would be useful.

## Figures and Tables

**Table 1:** ALK1 and endoglin inhibitors in clinical trials for cancer treatment.

Compound	Clinical Trial	Condition	Start Date	End Date (or Last Updated)	Status
PF-03446962	NCT01620970	Transitional Cell Carcinoma of Bladder	June 2012	May 2021	Completed
PF-03446962	NCT01911273	Hepatocellular Carcinoma	July 2013	October 2015	Terminated
Dalantercept	NCT01642082	Endometrial Adenocarcinoma Endometrial Adenosquamous Carcinoma Endometrial Mixed Adenocarcinoma Endometrial Mucinous Adenocarcinoma Endometrial Clear Cell Adenocarcinoma Endometrial Serous Adenocarcinoma Recurrent Uterine Corpus Carcinoma	July 2012	March 2018	Completed
Dalantercept	NCT01720173	Recurrent Fallopian Tube Carcinoma Recurrent Ovarian Carcinoma Recurrent Primary Peritoneal Carcinoma	November 2012	October 2021	Completed
Dalantercept	NCT00996957	Advanced Solid Tumors Multiple Myeloma	October 2009	March 2013	Completed
Dalantercept	NCT01458392	Squamous Cell Carcinoma of the Head and Neck	October 2011	June 2017	Completed
Dalantercept and Axitinib	NCT01727336	Advanced Renal Cell Carcinoma	November 2012	September 2021	Terminated (The study was terminated by the sponsor following unblinding of the Progression Free Survival endpoint)
Dalantercept plus Sorafenib	NCT02024087	Advanced Adult Hepatocellular Carcinoma	December 2013	March 2021	Completed
PF-03446962	NCT00557856	Advanced Solid Tumors	November 2007	October 2015	Completed
PF-03446962	NCT01337050	Neoplasms	April 2011	October 2015	Completed
PF-03446962	NCT01486368	Malignant Pleural Mesothelioma	December 2011	April 2020	Completed
PF-03446962 and Regorafenib	NCT02116894	Colorectal Cancer	April 2014	March 2019	Completed
TRC105 in combination with Bevazizumab	NCT01648348	Adult Anaplastic Astrocytoma Adult Anaplastic Oligodendroglioma Adult Giant Cell Glioblastoma Adult Glioblastoma Adult Gliosarcoma Adult Mixed Glioma Recurrent Adult Brain Neoplasm	July 2012	May 2018	Completed
TRC105 with Abiraterone and Enzalutamide	NCT03418324	Prostate Cancer	February 2018	December 2020	Completed
TRC105 in combination with Pazopanib.	NCT02979899	Advanced angiosarcoma	December 2016	May 2020	Completed
TRC105 in combination with Nivolumab	NCT03181308	Non small cell lung carcinoma	June 2017	June 2020	Completed
TRC105 in combination with Pazopanib	NCT01975519	Advanced Soft Tissue Sarcoma	November 2013	May 2020	Completed
TRC105 in combination with Bevazizumab	NCT01332721	Adult Solid Tumor	April 2011	December 2018	Completed
TRC105 in combination with Bevazizumab	NCT02664961	Gestational Trophoblastic Neoplasia Choriocarcinoma Placental Site Trophoblastic Tumor Epithelioid Trophoblastic Tumor	January 2016	July 2019	Terminated
TRC105 in combination with Sorafenib	NCT02560779	Hepatocellular Carcinoma	September 2015	July 2020	Completed
TRC105 in combination with Capecitabine	NCT01326481	Metastatic breast cancer	March 2011	March 2019	Completed
TRC105 in combination with Sorafenib	NCT01306058	Hepatoma Liver Neoplasms Adenoma Liver Cell Carcinoma, Hepatocellular Liver Neoplasms, Experimental	March 2011	January 2019	Completed

## Abbreviations

AMPK, AMP activated kinase; ALK1, activin receptor-like kinase-1; bFGF, basic fibroblast growth factor; BMP, bone morphogenetic protein; BMP9, bone morphogenetic protein-9; BMP10, bone morphogenetic protein-10 BMPRII, bone morphogenetic protein receptor II; CAF, cancer-associated fibroblast; CAM, chick chorioallantoic membrane; CD105, endoglin; CTGF, connective tissue growth factor; EC, endothelial cell; ECM, extracellular matrix; EGF, epidermal growth factor; EMT, epithelial–mesenchymal transition; GDF, growth differentiation factors; GDF, growth differentiation factor; HCC, hepatocellular carcinoma; HGF, hepatocyte growth factor; HHT, hereditary hemorrhagic telangiectasia; HUVEC, human umbilical vein endothelial cells; JNK, c-Jun N-terminal kinase; LLC, Lewis lung carcinoma; LPA, lysophosphatidic acid; MAPK, mitogen-activated protein kinase; MEF, mouse embryo fibroblasts; OS, overall survival; PAI-1, plasminogen activator inhibitor 1; PD-1, programmed cell death protein 1; PDGF, platelet-derived growth factor; PD-L1, programmed death-ligand 1; PD-L2, programmed death-ligand 2; PI3K/Akt, phosphatidylinositol 3-kinase/Akt; PlGF, placental growth factor; TGF-β, transforming growth factor β; TKI, Tyrosine kinase inhibitors; TME, tumor microenvironment; VEGFA, vascular endothelial growth factor-A.
